# Resilience and recovery in neurosurgical residency: Unpacking lessons from video game mechanics

**DOI:** 10.1016/j.bas.2023.101793

**Published:** 2023-08-14

**Authors:** Aaron Lawson McLean, Anna C. Lawson McLean

**Affiliations:** Department of Neurosurgery, Jena University Hospital – Friedrich Schiller University Jena, Jena, Germany

**Keywords:** Neurosurgery, Residency, Video games, Resilience, Wellness, Stress, DBNO, HoT, DoT

## Abstract

**Introduction:**

Neurosurgical residency is a challenging journey demanding cognitive acuity and resilience, mirrored strikingly in the dynamics of video gaming. Gaming concepts of Down-But-Not-Out (DBNO), Heal-over-Time (HoT), and Damage-over-Time (DoT) can serve as compelling analogues to elements of neurosurgical training.

**Material and methods:**

An innovative, cross-disciplinary methodology was implemented, blending elements of autoethnography, personal reflective narrative, and comprehensive literary review. The cornerstone of this approach was an experiential reflective analysis, where two neurosurgical residents critically examined the parallels between their residency experiences and video game mechanics, thereby applying a lens of heuristic introspection to their professional journey. Complementing this, a comprehensive narrative synthesis of existing literature on resilience, wellness, and stress in neurosurgical residency training was conducted.

**Results:**

The DBNO concept parallels the resilience demonstrated by neurosurgical residents, emphasizing the importance of a supportive network. The HoT concept, analogous to wellness practices, underscores the incremental restoration of energy necessary for maintaining stamina in neurosurgery training. The DoT concept symbolizes the need to manage the often insidious and deleterious effects of chronic stress on residents' wellbeing.

**Discussion and conclusion:**

These gaming concepts provide an integrative framework for understanding the challenges faced and strategies employed in neurosurgical residency. The interplay between resilience, wellness practices, and effective stress management, represented by DBNO, HoT, and DoT respectively, is critical for maintaining health and fostering professional excellence. By embedding these metaphors within the training paradigm, the neurosurgical residency journey can be navigated more effectively, promoting not only professional success but also personal growth and wellbeing.

## Highlights

• Video game metaphors can illuminate the neurosurgical residency journey.
• The Down-But-Not-Out (DBNO) concept may emphasize resilience in adversity.
• Heal-over-Time (HoT) may highlights the critical need for consistent wellness practices.
• Damage-over-Time (DoT) can signify the subtle yet impactful role of chronic stress.
• The integration of these concepts can enhance both personal growth and professional success in neurosurgical residency.

## 1 Introduction

Neurosurgical residency is a challenging journey, marked by steep learning curves, high-intensity experiences, and demanding expectations. Residents navigate a complex terrain that demands cognitive acuity, technical proficiency, emotional resilience, and consistent wellness practices (Bourne et al., 2013; Whitfield et al., 2023). These dimensions of neurosurgical training are critically intertwined, each shaping and being shaped by the others, forming a dynamic interplay that significantly impacts both the personal wellbeing and professional growth of the resident.

Within this intricate landscape, the world of video gaming offers an unexpected yet compelling parallel. Just as in neurosurgical training, success in the gaming arena depends on strategic decision-making, resilience under pressure, and the efficient management of resources over time (Despain and Acosta, 2013; Tichon and Mavin, 2016). Interestingly, certain gaming concepts such as Down-But-Not-Out (DBNO), Heal-over-Time (HoT), and Damage-over-Time (DoT) provide particularly potent metaphors for understanding the challenges and strategies associated with neurosurgical residency (Glossary of video game terms, 2023; Zubek, 2020).

In this paper, we explore these analogues, employing an innovative cross-disciplinary methodology that melds experiential reflective analysis with a comprehensive narrative synthesis of the literature. Through this approach, we aim to enhance understanding of the neurosurgical training experience, offering fresh perspectives on resilience and wellness, and ultimately informing strategies to foster both personal wellbeing and professional excellence within neurosurgical residency.

## 2 Materials and methods

We implemented a multi-faceted, cross-disciplinary approach that combined the personal narrative elements of autoethnography with a comprehensive narrative synthesis of existing literature (Wall, 2008). This methodology enabled a depth of examination that was at once both experientially grounded and academically rigorous.

The first stage of the methodology focused on experiential reflective analysis, wherein two neurosurgical residents intimately involved in the study provided a critical examination of their training experiences (Chang, 2008). Drawing from the tenets of autoethnography, they analyzed the intersection of their personal experiences and the broader cultural context of neurosurgical residency. This self-reflexive approach allowed the residents to explore the similarities between their residency experiences and video game mechanics, mapping their professional journey onto the corresponding gaming concepts (Popoveniuc, 2014). This personal exploration, grounded in the residents' lived experiences, offered an authentic insider perspective on the stressors and coping mechanisms integral to neurosurgical training.

The self-reflexive narratives served as a cornerstone for the second stage: a comprehensive narrative synthesis of the literature. Informed by the reflections derived from the residents' narratives, a thorough review of existing literature on resilience, wellness, and stress in residency training was conducted. This encompassed both quantitative and qualitative studies, broadening the scope to capture the multi-dimensionality of the resident experience. Employing a critical interpretive synthesis approach, diverse empirical findings were integrated, preserving nuanced understanding while extending the interpretation beyond the original context.

The self-reflexive narratives served as a cornerstone for the second stage: a comprehensive narrative synthesis of the literature. Informed by the reflections derived from the residents' narratives, a thorough review of existing literature on resilience, wellness, and stress in residency training was conducted. The literature search strategy was designed to retrieve a broad array of both quantitative and qualitative studies from several biomedical research databases, effectively capturing the multi-dimensionality of the resident experience. Our search strategy using the PubMed, EMBASE, and PsycINFO databases is outlined in a supplementary file (Supplementary File 1). Employing a critical interpretive synthesis approach, diverse empirical findings were integrated. This allowed for the preservation of nuanced understanding while extending the interpretation beyond the original context.

The combination of these two methodological pillars allowed for a robust exploration of the parallels between gaming mechanics and neurosurgical training dynamics. The resulting analysis thus produced a richly textured understanding that spanned personal experiences, the cultural milieu of residency, and the wider academic discourse.

## 3 Results

The innovative, cross-disciplinary methodology yielded a rich tapestry of data and insights. Through the autoethnographic analysis, the residents' narratives vividly illustrated the experience of neurosurgical training, elucidating the high-intensity moments and the resilience demanded in response to them. The reflective narratives underscored the importance of consistent wellness practices and highlighted the often insidious impact of chronic stress.

The literature review unearthed a broad range of quantitative and qualitative studies that further nuanced our understanding of resilience, wellness, and stress within the context of neurosurgical residency. Some common themes emerged across studies: the pivotal role of support systems, the importance of personal wellness strategies, and the potentially detrimental effects of chronic stress if left unmanaged.

Moreover, the distinct video game concepts – DBNO, HoT, and DoT – found their resonance in the narratives and empirical literature alike, providing compelling metaphors to frame the challenges and strategies associated with neurosurgical residency. These concepts not only enriched the interpretive process but also facilitated a unique dialogue between personal narratives, empirical literature, and gaming concepts, setting the stage for an integrated discussion.

## 4 Discussion

The journey through neurosurgical residency is a long and steep traverse, often marked by high-intensity experiences that demand both cognitive acuity and unwavering resilience (Mackel et al., 2021; Pevehouse, 1984). Its complexity, encompassing a multitude of professional challenges, personal growth phases, and inevitable stressors, finds an unexpected yet compelling parallel in the dynamic and high-stakes world of video gaming. In the realm of gaming, success hinges heavily on one's ability to demonstrate fortitude under pressure, employ strategic approaches in changing circumstances, and astutely balance immediate actions with long-term objectives – attributes that resonate strikingly with the neurosurgical resident's experience.

In recognizing these similarities, we find an invaluable opportunity to draw upon specific gaming concepts, such as DBNO, HoT and DoT, as conceptual tools to further our understanding of the neurosurgical residency landscape (*“Glossary of video game terms”*, 2023; Reynaldo et al., 2021). Each of these concepts carries distinct implications: DBNO underscores moments of high pressure and potential failure, where resilience is tested and the importance of support systems magnified; HoT represents consistent wellness practices, the steady recovery and replenishment of energy that is critical for enduring the demanding pace of neurosurgical training; DoT, on the other hand, symbolizes the insidious impact of chronic stress, subtly eroding a resident's wellbeing if not effectively managed (Table 1) (Asfaw et al., 2023; Klimo et al., 2013).

**Table 1:** Video game concepts and their relevance to neurosurgical residency. These concepts, woven into the fabric of a neurosurgical resident's journey, can offer an insightful perspective on the challenges faced and the strategies required to maintain health and wellbeing during this demanding period. Their understanding and application can be a crucial tool for residents and medical institutions alike.

Video Game Concept	Definition	Relevance to Neurosurgical Residency
Down-But-Not-Out (DBNO)	A near-defeat state in which a player is incapacitated but can be revived by teammates	Analogous to moments of high pressure or doubt in residency, highlighting the importance of resilience and support systems in residency
Heal-over-Time (HoT)	An effect that gradually restores a player's health over a period	Symbolizes the incremental restoration of physical and emotional health via wellness practices.
Damage-over-Time (DoT)	An effect that gradually reduces a player's health over time	Represents the insidious effect of chronic stress, which can quietly erode a resident's wellbeing if not managed effectively

In light of Stienen et al.‘s findings, which highlight the variable nature of theoretical and practical aspects of neurosurgical training across European countries, the need for adaptable strategies to foster resilience and wellness becomes apparent (Stienen et al., 2015). Understanding this variability and its impact on resident experiences further underscores the relevance of our gaming concepts, as they provide a flexible framework to navigate the distinct challenges and stressors encountered in diverse training environments.

### 4.1 DBNO and resident resilience: standing strong amidst the storms

In the gaming world, a player temporarily incapacitated but capable of recovery is in the DBNO state. Neurosurgical residents, akin to DBNO players, may experience moments of incapacitation, exhaustion and self-doubt in response to the manifold challenges presented by their training, such as mastering complex surgical procedures, acclimating to intense on-call rotations, high patient acuity, or navigating difficult patient outcomes (Zaed et al., 2020, 2022). Nevertheless, resilience emerges as a key trait, fueled by a supportive network of mentors, colleagues, family and friends, and personal coping mechanisms (Table 2) (Neal and Lyons, 2020; Shakir et al., 2020). Just like a DBNO player, the resident is not defeated and can be revived through the support of their team, both inside and outside the hospital.

**Table 2:** Factors impacting resilience in neurosurgical residency.

Factors	Description
Family and Social Support	Strong relationships outside the hospital environment provide emotional support and a sense of balance.
Operating Room Dynamics, Professional Cohesion	A supportive, respectful operating environment can bolster confidence and resilience. Positive interactions with surgical teams, nurses, and anesthesiologists contribute to resilience.
Personal Coping Mechanisms	Exercise, mindfulness practices, and hobbies can aid in stress management and mental rejuvenation.
Mentorship	Guidance and support from experienced surgeons can offer valuable perspective and advice.

### 4.2 HoT and sustaining stamina: winning the marathon

In a video game, a character's health is gradually restored through HoT effects. This concept is a metaphor for the importance of incremental, consistent health restoration practices in a neurosurgical residency. Amidst grueling surgical schedules and intense cognitive demands, consistent wellness practices – that restore energy, focus, and emotional equilibrium over time – provide the stamina needed to succeed. Whether it is to overcome a day-long complex spinal surgery or in navigating the nuanced trajectories of neurocritical care patients, a resident, like a gaming character, requires an effective health restoration strategy.

In maintaining wellness throughout neurosurgical residency, several key practices emerge as notably beneficial. Regular exercise, for instance, plays a pivotal role in enhancing stamina and managing stress, bolstering resilience in the face of the demanding residency environment. Nutritious dietary habits serve as another cornerstone, providing the sustained energy required during prolonged surgical procedures and arduous on-call duties. Adequate sleep is also paramount, not just in terms of restoring physical energy but more crucially for maintaining cognitive function, ensuring surgical precision, and fostering emotional regulation. Finally, incorporating mindfulness techniques can also prove to be invaluable, promoting mental clarity and effective stress management – critical attributes for navigating the complex landscape of neurosurgical residency (Asfaw et al., 2023; Berardo et al., 2020; Fargen et al., 2016; Spiotta et al., 2019; Wolfe et al., 2019). These core practices form the backbone of a robust wellness strategy for neurosurgical residents, underscoring the HoT concept.

### 4.3 DoT and chronic stress: the silent adversary

The DoT concept, a signature feature of many video games that depicts a slow, gradual decrease in a player's health points, serves as a striking metaphor for the insidious and often underestimated effect of chronic stress in neurosurgical residency. In the demanding landscape of neurosurgery, residents frequently contend with extensive working hours, the immense responsibility of decision-making, and the profound emotional burden associated with patient outcomes (Asfaw et al., 2023; Klimo et al., 2013; Shah et al., 2022; Shakir et al., 2018). Each of these factors incrementally contributes to a chronic stress load that, akin to the DoT effect, subtly and persistently erodes a resident's psychological and physical wellbeing.

The ramifications of such chronic stress extend beyond immediate wellbeing and are implicated in more severe long-term outcomes, such as resident burnout and other mental health disorders. Furthermore, it's worth acknowledging that this stress also has a potential effect on a resident's professional performance, potentially impacting their ability to deliver high-quality patient care (Kelly et al., 2021; Mackel et al., 2021; Zaed et al., 2020). As in the gaming world, where an unchecked DoT effect can imperceptibly deplete a player's health to critical levels, unmanaged chronic stress can steadily drain a resident's resilience and vitality, often with only subtle warning signs. Thus, understanding, acknowledging, and adequately addressing chronic stress – the DoT of neurosurgical residency – is an imperative aspect of maintaining health and performance throughout residency and beyond into the post-residency career stage.

### 4.4 Navigating the landscape of neurosurgical residency: an integrated approach

The DBNO, HoT, and DoT concepts, when woven together, form an integrated blueprint for maintaining health and wellbeing during neurosurgical residency. The interplay of these factors is crucial: resilience (DBNO) can be tested in high-pressure moments, such as an unexpected complication in surgery or a demanding on-call night. However, daily wellness practices (HoT) provide a steadying influence, helping to restore energy and focus. Meanwhile, identifying and managing chronic stressors (DoT) is essential to prevent the silent erosion of wellbeing.

### 4.5 Further work

Drawing from recent international survey data from medical students, residents, and recent graduates that sought to identify barriers to neurosurgical training in high- and low-to middle-income countries, it becomes apparent that the challenges within neurosurgical training are not confined to resilience and wellbeing but also include broader issues of global access and equity (Garba et al., 2022; Marchesini et al., 2022; Sarpong et al., 2022). These findings underscore the importance of our endeavor to understand and ameliorate the experience of neurosurgical residents, potentially extending the beneficial implications of our gaming analogy-based interventions to a global scale.

Recognizing the focus on wellness, the future of our video game mechanics approach could be strengthened by adopting elements from artificial intelligence (AI) in surgical simulations, as elucidated by Park et al. (2022). AI, when used in surgical simulations, can not only improve technical competence, but also enhances personal evaluation and feedback mechanisms which could be pivotal in managing the stress (DoT) faced by neurosurgical residents. Additionally, AI's potential to create personalized, adaptive learning environments can align with our HoT concept, facilitating continuous growth, resilience (DBNO), and wellness practices (Kabudi et al., 2021). The integration of AI could further our understanding of the intricate balance between resilience, wellness, and stress, and provide innovative tools to enhance wellbeing in neurosurgical residency.

## 5 Conclusion

Neurosurgical residency, much like the fast-paced, dynamic world of video gaming, is a space of continual adaptation, quick decision-making, and resilience against formidable odds. In this context, gaming concepts such as DBNO, HoT, and DoT can offer compelling metaphors for the challenges and strategies associated with neurosurgical residency. By integrating these lessons into the fabric of neurosurgical training, both at an individual and institutional level, we can cultivate an environment that nurtures not only the development of excellent surgeons but also the preservation of their health and wellbeing. The voyage through neurosurgical residency, thus, becomes not merely a test of endurance but a triumph of personal growth and professional excellence.

## Funding

This research was conducted independently without any external funding or financial support.

## Authorship

Both authors contributed equally to the research, development and drafting of this manuscript. Both authors approved the final version and confirm that they meet all International Committee of Medical Journal Editors criteria for authorship.

## Declaration of competing interest

The authors declare that they have no known competing financial interests or personal relationships that could have appeared to influence the work reported in this paper.
