# Supplementary material for: Resilience and recovery in neurosurgical residency: Unpacking lessons from video game mechanics
Source: Brain Spine. 2023 Aug 14;3:101793. doi: 10.1016/j.bas.2023.101793 (PMC10668065; doi:10.1016/j.bas.2023.101793)
Supplement: Multimedia component 1 [file mmc1.docx]

**Supplementary File 1: Literature Search Strategy**

**Database: PubMed**

Search Terms: ("resilience"[All Fields] OR "wellness"[All Fields] OR "stress"[All Fields]) AND ("residency"[All Fields] OR "training"[All Fields]) AND "neurosurg*"[All Fields]

Search Date:

**Database: EMBASE**

Search Terms: ('resilience' OR 'wellness' OR 'stress') AND ('residency' OR 'training') AND 'neurosurg*'

**Database: PsycINFO**

Search Terms: (resilience OR wellness OR stress) AND (residency OR training) AND neurosurg*

For all searches, no date restrictions were applied, and only articles in English were considered. All searches were performed on 24^th^ May 2023. Articles were screened by title and abstract for relevance. The reference lists of selected articles were also reviewed to identify further relevant studies. This comprehensive search strategy ensured a robust and extensive coverage of the literature relating to resilience, wellness, and stress in neurosurgical residency.
